# Pharmacological TRPC6 inhibition improves survival and muscle function in mice with Duchenne muscular dystrophy

**DOI:** 10.1172/jci.insight.158906

**Published:** 2022-10-10

**Authors:** Brian L. Lin, Joseph Y. Shin, William P.D. Jeffreys, Nadan Wang, Clarisse A. Lukban, Megan C. Moorer, Esteban Velarde, Olivia A. Hanselman, Seoyoung Kwon, Suraj Kannan, Ryan C. Riddle, Christopher W. Ward, Steven S. Pullen, Antonio Filareto, David A. Kass

**Affiliations:** 1 Department of Cardiology,; 2 Department of Orthopaedics, and; 3 Department of Radiation Oncology and Molecular Radiation Sciences, Johns Hopkins University, Baltimore, Maryland, USA.; 4 Department of Orthopedics, University of Maryland, Baltimore, Maryland, USA.; 5 Cardiometabolic Diseases Research and; 6 Research Beyond Borders, Boehringer Ingelheim Pharmaceuticals, Ridgefield, Connecticut, USA.; 7 Department of Pharmacology and Molecular Sciences, Johns Hopkins University, Baltimore, Maryland, USA.

**Keywords:** Cardiology, Muscle Biology, Calcium channels, Cardiovascular disease, Muscle

## Abstract

Gene mutations causing loss of dystrophin result in the severe muscle disease known as Duchenne muscular dystrophy (DMD). Despite efforts at genetic repair, DMD therapy remains largely palliative. Loss of dystrophin destabilizes the sarcolemmal membrane, inducing mechanosensitive cation channels to increase calcium entry and promote cell damage and, eventually, muscle dysfunction. One putative channel is transient receptor potential canonical 6 (TRPC6); we have shown that TRPC6 contributed to abnormal force and calcium stress-responses in cardiomyocytes from mice lacking dystrophin that were haplodeficient for utrophin (*mdx/utrn^+/–^* [HET] mice). Here, we show in both the HET mouse and the far more severe homozygous *mdx/utrn^–/–^* mouse that TRPC6 gene deletion or its selective pharmacologic inhibition (by BI 749327) prolonged survival 2- to 3-fold, improving skeletal and cardiac muscle and bone defects. Gene pathways reduced by BI 749327 treatment most prominently regulated fat metabolism and TGF-β1 signaling. These results support the testing of TRPC6 inhibitors in human trials for other diseases as a novel DMD therapy.

## Graphical abstract

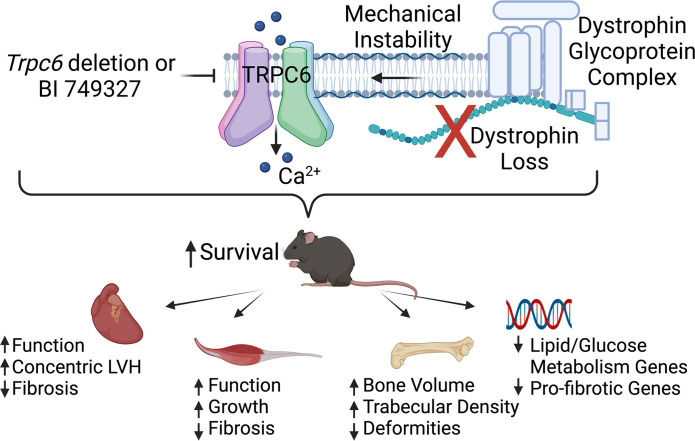

## Introduction

Duchenne muscular dystrophy (DMD) is an X-linked disorder affecting approximately 0.02% live male births; it is caused by gene mutations in the cytoskeletal macromolecule dystrophin that lead to negligible expressed protein**)** (1). Dystrophin is critical to striated muscle integrity and function, and patients with DMD experience severely decreased mobility by their early teens, with marked kyphoscoliosis, cardiomyopathy, and death by the second to fourth decade (2, 3). Lack of dystrophin alters sarcolemmal membrane mechanical stability and signaling, disrupting Ca^2+^ homeostasis. The latter has been linked to oxidative and nitrosative stress and muscle degeneration as well as exacerbation of disease progression and severity (4–7). Muscle-targeted therapy includes corticosteroids, and several exon skipping approaches have been approved (8–10), though overall effect remains limited. Microdystrophin (11) and gene editing (12, 13) efforts are also being pursued, but these remain investigational. Notably, small-molecule treatment for DMD remains sorely lacking.

Increased intracellular calcium (Ca^2+^_i_) linked to mechanical perturbation is among the cellular mechanisms linked to DMD muscle dysfunction (14). Studies first proposed that elevated Ca^2+^_i_ results from membrane disruption (6), although other studies found Ca^2+^ influx to be mediated by cation channels (4, 14, 15). One such channel is transient receptor potential canonical 6 (TRPC6), a mechanosensitive nonvoltage gated cation channel that primarily conducts calcium (16). TRPC6 is found at low expression levels and activity in many cell types under normal physiological conditions, and mice lacking *Trpc6* have minimal phenotype (4, 17, 18). However, the channel is activated notably by GPCR receptor–coupled signaling via diacylglycerol (19), and it has also been linked to mechanical stress (16). TRPC6 gain-of-function mutations in humans are a cause of focal segmental glomerulosclerosis with abnormal calcium homeostasis and podocyte function (20). TRPC6 expression also increases in pressure-overloaded hearts (21), and genetic overexpression in cardiomyocytes induces heart failure (22). Overexpression of a dominant-negative TRPC6 in skeletal muscle of *mdx* mice mitigates histopathological changes (15).

TRPC6 gene and protein levels are also elevated in cardiac and skeletal muscle in mice (4, 17, 23) and humans (24, 25) with DMD (Supplemental Figure 1A; supplemental material available online with this article; https://doi.org/10.1172/jci.insight.158906DS1). Moreover, in cardiac muscle and myocytes from DMD mice lacking dystrophin and haplodeficient for utrophin (*mdx/utrn^+/–^* [HET] mice), TRPC6 mediates an abnormal rise in Ca^2+^_i_ induced by acute mechanical stress, resulting in excessive force generation and arrhythmia (4). These abnormalities are suppressed by *Trpc6* gene deletion or acute inhibition with a small-molecule antagonist (4). While this antagonist could not be tested in vivo due to rapid clearance, another potent and selective TRPC6 inhibitor — BI 749327 — has since been developed with oral bioavailability and pharmacology suitable for in vivo use (21). A variant of this molecule has already been studied in phase I and II clinical trials, including in healthy male adults (NCT04665700 and NCT03854552), for renal dysfunction (NCT04176536), and for acute severe COVID-19 (NCT04604184).

This study tested the efficacy of chronic TRPC6 suppression by BI 749327 in mice lacking both dystrophin and utrophin (*mdx/utrn^–/–^* [DKO] mice), which represent a severe DMD model (26, 27) as well as in HET mice. We compared these results with those from DKO mice lacking *Trpc6*. We found that blocking TRPC6 prolonged survival in DKO and HET mice, improving skeletal and cardiac morphology and dysfunction, and reducing skeletal and bone deformities.

## Results

### DKO mice lacking Trpc6 showed improved survival, muscle function, and bone deformities.

**Figure 1:**
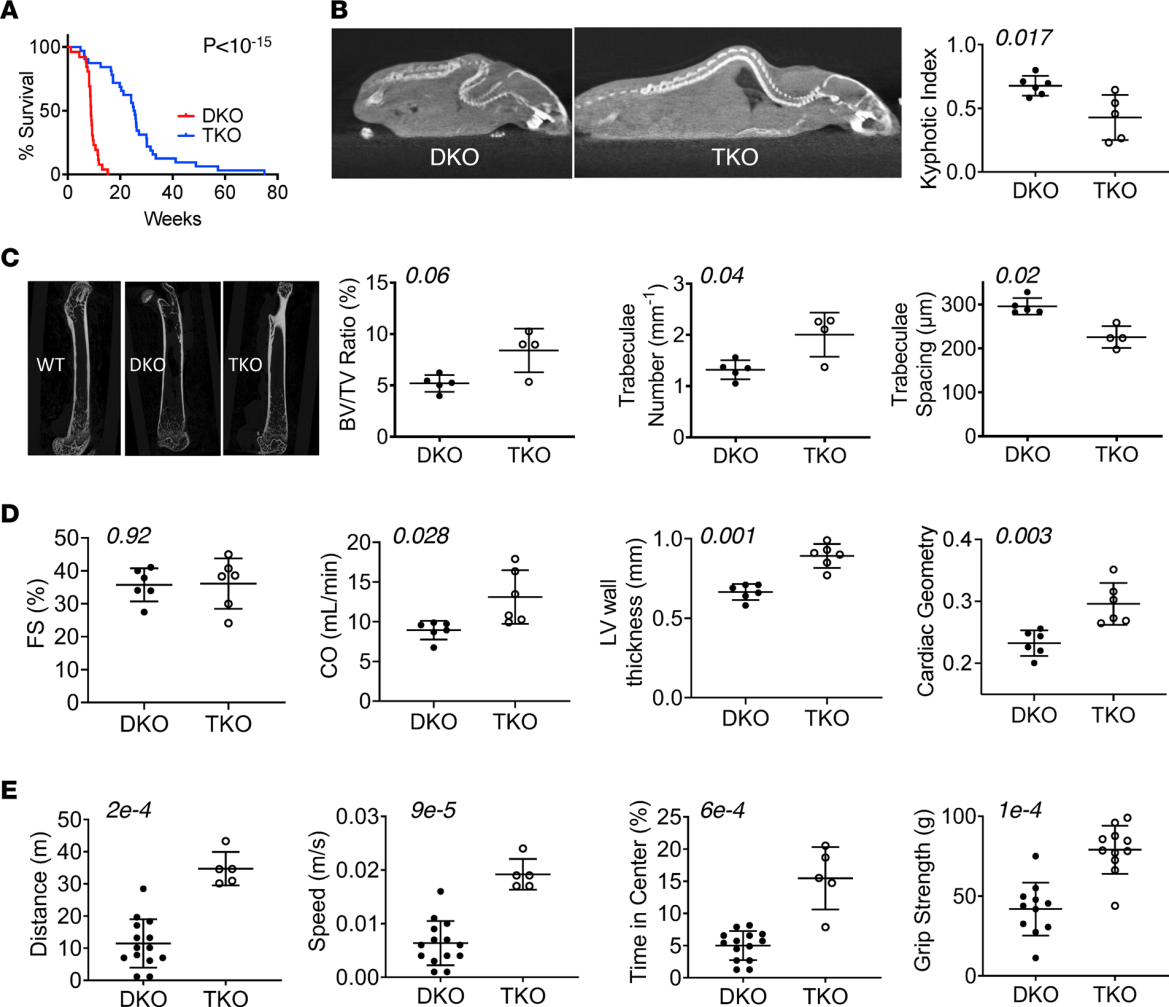
TRPC6 gene deletion in the *mdx/utrn^–/–^* DKO mouse model of DMD improves dystrophic phenotype. (**A**) Survival curves for DKO mice versus TKO mice (*n =* 26 DKO, *n =* 32 TKO). (**B**–**E**) Data are from animals at approximately 8 weeks of age. (**B**) Computerized tomography image example, and summary results for spinal kyphosis in DKO compared with TKO mice. (**C**) Left*:* Example microCT images of femurs. Right*:* Summary data for the bone volume/tissue volume (BV/TV) ratio, trabeculae number, and trabeculae spacing (*n =* 5 DKO, *n =* 4 TKO). (**D**) Echocardiography of conscious mice for fractional shortening (FS), cardiac output (CO), average left ventricular (LV) wall thickness, and cardiac geometry (LV thickness/cross-sectional diameter ratio) (*n =* 6 DKO, *n =* 6 TKO). (**E**) Open-field test results for voluntary movement distance, speed, time spend in the center of the field (*n =* 14 DKO, *n =* 5 TKO), and forelimb grip strength (*n =* 11/group). (**A**) Log-rank test; (**B**, **C**, and **E**) Mann-Whitney *U* test; (**D**) unpaired Student’s *t* test. *P* values are shown in each panel.

DKO mice had a median survival of approximately 9 weeks, with 100% mortality by 4 months. DKO mice also lacking *Trpc6* (TKO) had a near 3-fold increase in median survival (*P <* 10^–15^, Figure 1A). Age-matched TKO mice also had greater total body, heart, lung, and skeletal muscle mass compared with DKO mice (Supplemental Figure 1B). DKO mice developed marked kyphosis that was also reduced in TKO mice (Figure 1B). *Trpc6* deletion improved bone structure, with a greater bone tissue volume/total tissue volume ratio and trabeculae number and reduced intertrabecular spacing to levels that were closer to those of WT controls (Figure 1C and Supplemental Table 1A). Fractional shortening was not significantly changed, and while cardiac output was increased (Figure 1D), this was due to faster heart rate and not greater stroke volume (Supplemental Table 2A). The cardiac left ventricular (LV) wall thickness and geometric ratio (diastolic LV wall thickness/interior dimension ratio) rose in TKO mice, consistent with concentric hypertrophy. Mobility, as measured in the open-field test, improved in TKO mice, with a greater average distance and speed walked and more time spent in the field center, reflecting greater motor confidence, compared with DKO mice (Figure 1E and Supplemental Video 1). TKO mice also exhibited increased forelimb grip strength. These changes also narrowed disparities between TKO and WT mice (Supplemental Table 1A). We also assessed HET mice lacking *Trpc6* and found they too had reduced mortality compared with HET mice expressing *Trpc6* (Supplemental Figure 2). Together, these data show improved survival and amelioration particularly of skeletal muscle and bone defects from *Trpc6* deletion in DKO mice.

### BI 749327 ameliorates the DMD phenotype in both DKO and HET mice.

**Figure 2:**
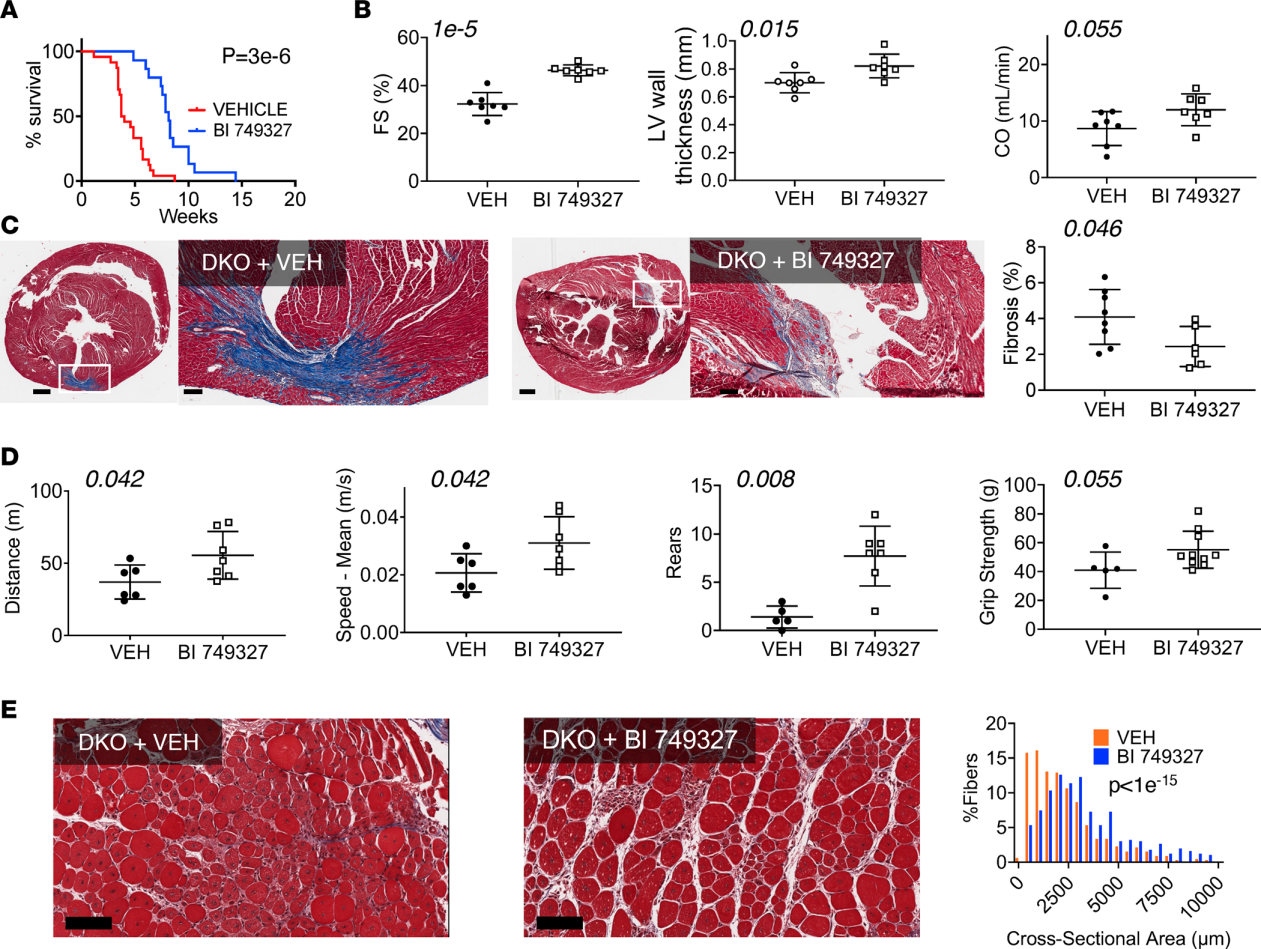
TRPC6 blocker BI 749327 extends survival, improves striated muscle function, and reduces dystrophic histopathology in DKO mice. (**A**) Survival curves for DKO mice with BI 749327 treatment or placebo (vehicle) treatment (*n =* 24 vehicle, *n =* 15 BI 749327; log-rank test). (**B**–**E**) Data are from animals at approximately 6 weeks of age from each group. (**B**) Echocardiography: fractional shortening (FS), cardiac output (CO), and left ventricular (LV) thickness (*n =* 7 vehicle [VEH], *n = 7* BI 749327; unpaired *t* test). (**C**) Left and middle*:* Example 6-week Masson’s trichrome stains of whole heart cross sections, with high-magnification of region focal fibrosis. Scale bar: 500 μm (first and third image); 100 μm (second and fourth image). Right*:* Summary analysis (*n =* 8 vehicle, *n =* 6 BI 749327; unpaired *t* test). (**D**) Integrated skeletal motor function assessed by open-field test distance and speed (*n =* 6 vehicle, *n =* 7 BI 749327; unpaired *t* test), rearing behavior (*n =* 5 vehicle, *n =* 7 BI 749327; Mann-Whitney *U* test), and grip strength (*n =* 5 vehicle, *n =* 10 BI 749327; Mann-Whitney *U* test). (**E**) Example histology and summary distribution of cross-sectional area of muscle fibers in gastrocnemius (*n =* 659 vehicle, *n =* 563 BI 749327 fibers, *n =* 3 animals/group; Mann-Whitney *U* test). Scale bar: 100 μM.

To test the effect of pharmacological TRPC6 inhibition, BI 749327 or vehicle (placebo) was administered subcutaneously, starting on P3. Pharmacokinetics confirmed stable drug levels throughout a 24-hour period (data measured 1 and 24 hours after AM dosing; Supplemental Figure 2B). Median survival time in placebo-treated DKO mice was shorter than that in the first study. However, placebo-treated DKO mice were handled and injected daily, and such stress can shorten survival (28, 29). Importantly, despite similar stresses, DKO mice receiving BI 749327 had nearly double the life span of DKO mice receiving vehicle (Figure 2A). Due to more rapid mortality, functional measurements were taken at 6 weeks of age (versus 8 weeks in the prior study; c.f. Figure 1). In contrast to genetic KO in the TKO mice, TRPC6 inhibition with BI 749327 increased fractional shortening (*P =* 10^–5^) and ejection fraction (*P =* 0.0004) (Figure 2B and Supplemental Table 2B). Cardiac output trended higher, not because of an increase in heart rate, but because of increased stroke volume with reduced end-systolic volume at a similar preload (Supplemental Table 2B). TRPC6 inhibition also led to concentric LV remodeling (Figure 2B and Supplemental Table 2B). There was modest trend to reduced myocardial fibrosis quantified in whole heart sections (Figure 2C). Open-field tests also suggested improved movement with BI 749327 treatment, and while there was more overlap between groups than in the *Trpc6* gene deletion study, both distance and speed of movement still improved to be closer to that of WT controls (Supplemental Table 1B and Supplemental Video 2). Rearing was more frequent and grip strength also had a rising trend in drug-treated DKO mice (Figure 2D). Skeletal muscle fiber cross-sectional area increased in gastrocnemius, soleus, and tibialis anterior muscles (Figure 2E and Supplemental Figure 3, A and B), with a trend of less fibrosis in the gastrocnemius muscle (Supplemental Figure 3C). Together, these data support improved cardiac and skeletal muscle function in DKO mice treated with BI 749327.

**Figure 3:**
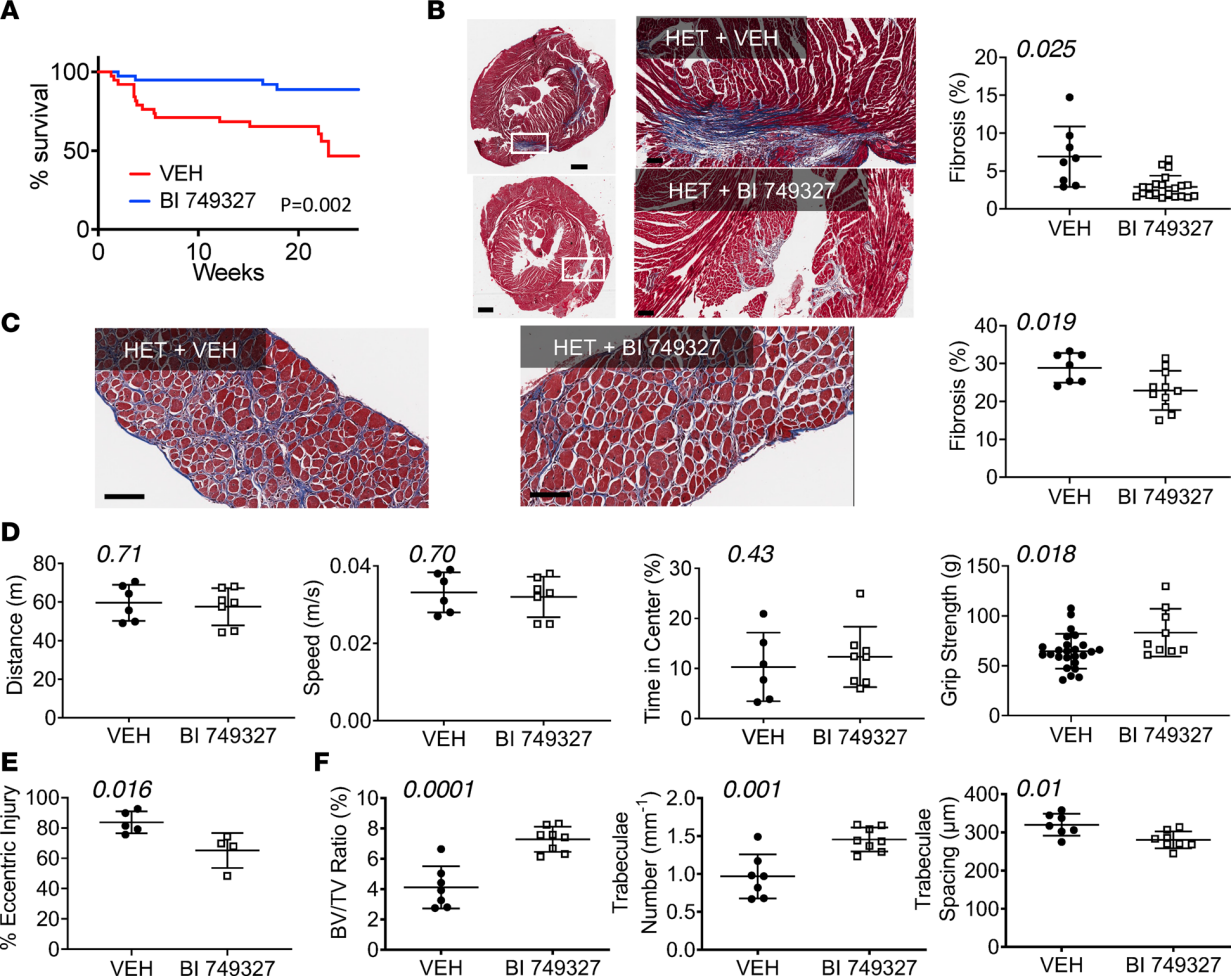
BI 749327 extends survival, improves striated muscle function, bone structure, and dystrophic histopathology in HET DMD mice. (**A**) Survival curves for HET mice treated with BI 749327 or vehicle (*n =* 38 vehicle, *n =* 39 BI 749327; log-rank test). (**B**–**F**) Data are from mice at approximately 12 weeks of age. (**B**) Left*:* Example Masson’s trichrome–stained left ventricle cross sections at low and high magnification, as in Figure 2C. Scale bar: 500 μm (left); 100 μm (right). Right*:* Summary results for fibrosis quantitation (*n =* 8 vehicle, *n =* 21 BI 749327; Welch’s unpaired *t* test). (**C**) Diaphragm was stained as in **B** and quantification of fibrosis (*n =* 7, *n =* 11; Student’s unpaired *t* test). Scale bar: 100 μm. (**D**) Open-field testing distance, speed, and time spent in center of the field (*n =* 6 vehicle, *n =* 7 BI 749327; data at 12 weeks of age), and grip strength (*n =* 9 vehicle, *n =* 7 BI 749327; data at 6 weeks of age). All analyzed with Student’s unpaired *t* test. (**E**) Eccentric injury determined in gastrocnemius muscle induced by sequential tetanic contractions in anesthetized HET mice treated with vehicle or BI 749327. Force of the last contraction was normalized by that for the initial contraction to assess relative eccentric injury (*n =* 5 vehicle, *n =* 4 BI 749327; Mann-Whitney *U* test) in 20-week-old mice. (**F**) Femur bone/tissue volume ratio, trabeculae number, and trabeculae spacing (*n =* 7 vehicle, *n =* 8 BI 749327; Student’s *t* test) at 12 weeks age.

BI 749327 improved survival in HET DMD mice as well (*P =* 0.002, Figure 3A), reducing fibrosis in the myocardium (Figure 3B) and diaphragm (Figure 3C). There was no significant change in cardiac parameters (Supplemental Figure 4A and Supplemental Table 2C), although placebo-treated HET mice themselves had minimal abnormalities. Open-field mobility was also unchanged with treatment (Figure 3D). BI 749327 treatment improved grip strength (Figure 3D) and reduced eccentric muscle injury (Figure 3E and Supplemental Figure 4B). We also found a greater bone tissue volume ratio, increased trabeculae number and thickness, and tighter trabecular spacing in femurs in HET mice treated with BI 749327, compared with mice treated with vehicle (Figure 3F and Supplemental Figure 4, C and D). Thus, TRPC6 inhibition was able to improve some features of the milder HET DMD phenotype.

### Effect of Trpc6 gene deletion or BI 749327 on cardiac transcriptome of DKO mice.

**Figure 4:**
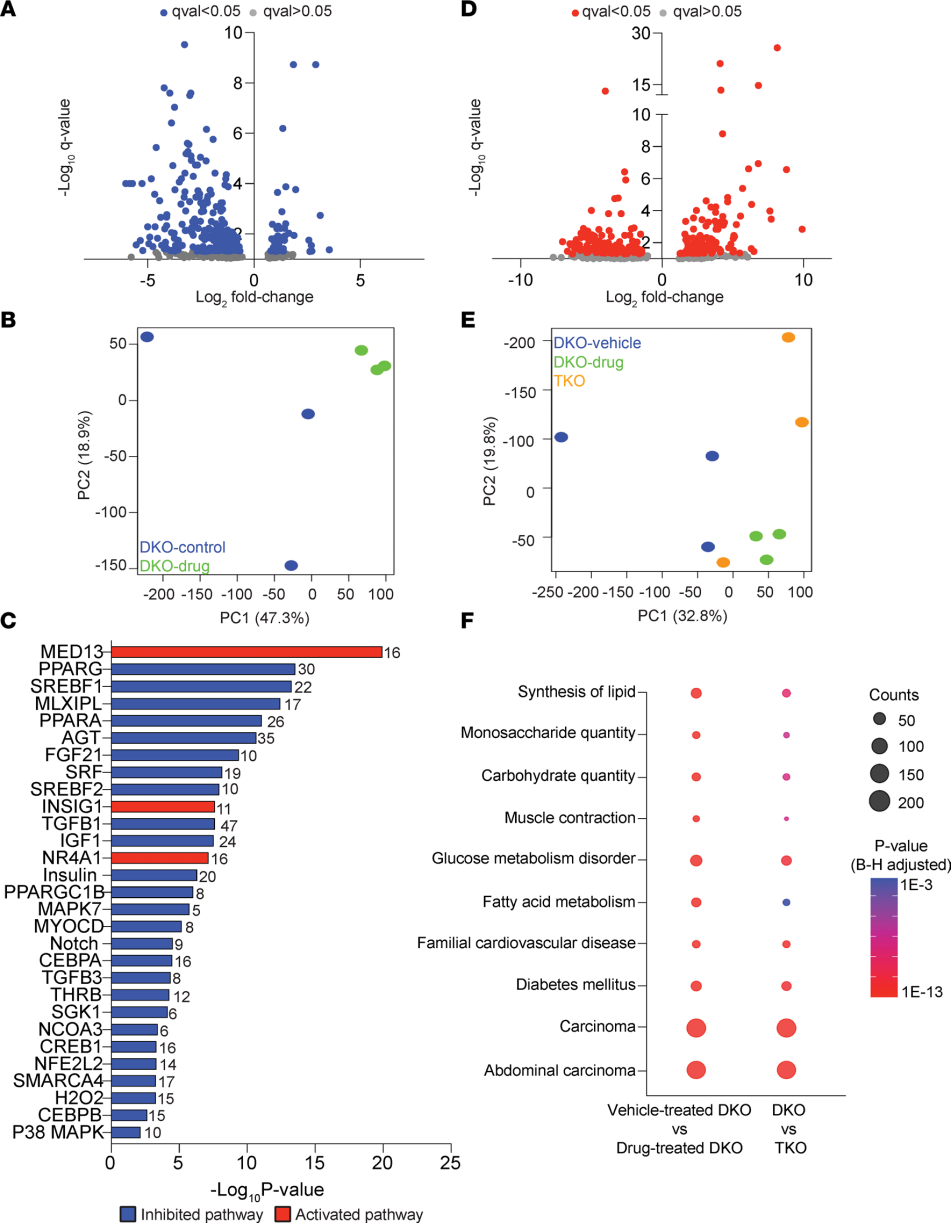
Transcriptome analysis of BI 749327–treated DKO mice reveals significantly reduced expression of genes involved in lipid synthesis and fibrosis pathways. (**A**) Volcano plot of differentially expressed genes in BI 749327– and vehicle-treated DKO mice (*n =* 3 biological replicates, female DKO mice, 8 weeks of age; FDR < 0.05). (**B**) PCA analysis of the same data revealed a treated DKO cluster separated from vehicle controls. (**C**) Ingenuity transcription regulatory analysis of proximal signaling pathways (all with an absolute activation *z* score > 2). Dominant downregulated pathways involve lipid and carbohydrate metabolism and fibrosis. (**D**) Volcano plot for differentially regulated genes in TKO and DKO mice, showing a similar number of upregulated and downregulated genes, with broader log fold changes than with the drug intervention. (**E**) PCA plot comparing DKO and TKO mice as well as DKO mice treated with BI 749327, showing separation of both from DKO mice in principal component 1 (PC1). (**F**) Ingenuity pathway analysis of differentially downregulated pathways identified many significant pathways shared by BI 749327–treated compared with vehicle-treated DKO mice as well as DKO mice compared with TKO mice. Dot size indicates the number of genes differentially altered in the pathway, and color indicates the *P* value. Shared pathways were also enriched for lipid and carbohydrate metabolism.

The myocardial molecular signature altered by BI 749327 treatment or genetic *Trpc6* deletion was examined by RNA-Seq of LV myocardium. Figure 4A displays a volcano plot comparing differential gene expression for DKO mice with and without BI 749327 treatment. There were approximately 260 differentially expressed genes downregulated by BI 749327. Principal component analysis (PCA) (Figure 4B) showed clustering of drug-treated DKO mouse factors away from those of vehicle-treated DKO mice. Ingenuity transcription regulatory pathway analysis found many downregulated factors controlling lipid metabolism (e.g., MED13, PPARA and PPARG, SREBF1 and SREBF2, INSIG1, PPARGC1B), glucose uptake and signaling (e.g., insulin and IGF1), and fibrosis (e.g., FGF21, SRF, TGFB1) (Figure 4C). We also examined differentially expressed genes between the TKO and DKO models; we found about the same number as with the drug intervention (Figure 4D). PCA showed separation in principal component 1 of these data from those of DKO-treated vehicle controls (Figure 4E). Similar transcription regulatory analysis of TKO versus DKO mice differed from the drug treatment comparison, yielding mostly upregulated pathways associated with growth (e.g., MRTFA, EGF, TEAD3, MEF2C, STAT3, PDGF) (Supplemental Figure 5). However, we still found shared enriched disease pathways with both TRPC6 drug inhibition or *Trpc6* gene deletion that involved mostly lipid and glucose metabolism (Figure 4F); full gene lists for each are provided in Supplemental Table 3.

## Discussion

This study reveals chronic suppression of TRPC6 with a selective small-molecule inhibitor ameliorates DMD pathobiology in cardiac and skeletal muscle and improves survival. These effects are analogous, though not entirely replicative, to those achieved by embryonic *Trpc6* gene deletion. Importantly, we tested the treatment in the DKO mouse, one of the more severe models of DMD. While other therapies have been shown to improve the DKO phenotype (30, 31), few have reported enhanced survival and each used genetic manipulations (overexpression or knockdown) (32–35). To our knowledge, the current study is the first to demonstrate survival efficacy from a small-molecule therapy. That we also observe benefit without replacing dystrophin or utrophin suggests that TRPC6 plays an important downstream role in DMD disease. While we do not suggest that this is likely to be superior to dystrophin replacement, inhibiting TRPC6 may well find utility itself and/or in combination with genetic strategies. Importantly, a member of the same TRPC6 inhibitor class has been tested in humans (healthy men, NCT04665700, NCT03854552; renal disease, NCT04176536; and COVID-19, NCT04604184), enhancing the translational significance of our findings.

There are a few differences between the results obtained in the experiments comparing DKO mice with TKO mice and those comparing DKO mice with or without BI 749327. Both control groups are composed of DKO mice: the first group was left alone in their cages until studied, the latter group received daily handling after P3, with subcutaneous injections. As noted, stress from daily handling may contribute to more rapid mortality, and mortality in the similarly handled control HET mice, which is rarely observed in this milder phenotype, further supports this hypothesis. However, other features differed that would seem in conflict with the mortality results. For example, the open-field test distance and speed were greater in DKO controls that had been handled daily (required for drug administration), yet these same animals had earlier mortality than unhandled DKO controls. Some of this difference may relate to the age of the mice; handled DKO mice were 25% younger. Age differences can be important to this type of testing (36). Alternatively, mice handled daily that survived to have the open-field testing performed better due to behavioral effects of the daily human/animal interaction (37, 38). While some drift in our genetic lines is another possibility, we suspect this was not major factor, as cohorts generated over several years were combined for many of these metrics.

While a role for TRPC6 in hearts and cardiomyocytes lacking dystrophin/utrophin has been previously reported (23, 25, 39), data in DMD skeletal muscle has been previously lacking. Here, we found that both gene deletion and small-molecule TRPC6 inhibition improved muscle morphology and function in DKO and HET mice, accompanied by enhanced bone structure. Increased grip strength could reflect greater muscle mass, supported by increased fiber diameter in several muscle groups and overall body weight. While we did not measure body weight in most DKO mice, we did in HET mice, and grip strength/body weight did not significantly change with BI 749327 treatment (Supplemental Figure 4E). Thus, integrated motor performance was likely tied to increased muscle and overall body mass. The bone defects that we observed in DKO mice are also found in human DMD (40), yet we have not had methods to improve bone defects in DMD. Their diminution with TRPC6 inhibition could result from increased muscle growth and force. Both dystrophin and TRPC6 are also expressed in vascular smooth muscle, and in *mdx* mice TRPC6 contributes to greater Ca^2+^_i_ in vessels exposed to cyclic stretch, leading to cell damage (23). Finally, TRPC6 is also present in immune cells, including neutrophils, where TRPC6 regulates activation by CXCL1 (41) and lymphocytes to promote apoptosis (42). TRPC6 upregulation contributes to endothelial permeability and cell diapedesis of immune cells by loosening cell-cell junctions (43). Thus, there are various cell types that modulate DMD that could be offset by TRPC6 inhibition to enhance muscle function.

In addition to TRPC6, close family members TRPC1 and TRPC3 are also expressed in cardiac and skeletal muscle, and DMD studies have found that each can play a role in excessive mechanoactivated Ca^2+^ entry (4, 15, 44, 45). Genetic TRPC3 upregulation in skeletal muscle induces a DMD-like phenotype in WT mice (15), although, in the heart, TRPC3 does not regulate altered calcium/force mechanosignaling in DMD (4). Whether chronic TRPC3 or TRPC1 inhibition modifies DMD in vivo has not been reported. TRPC proteins generally appear as heterotetramers (16), with TRPC6 notably associating with TRPC1 and TRPC3. Thus, a TRPC6 inhibitor likely indirectly restricts these other proteins as well acting as a poison peptide, whereas targeted *Trpc6* deletion leaves them available to form alternative functional channels. We previously examined expression of all 3 TRPC channels in HET DMD (4); we found that only TRPC6 was upregulated and found no upregulation of the other channels in TRPC6-KO mice (17).

It remains uncertain how the lack of dystrophin and associated dystro-sarcoglycan complex results in a pathobiological role for TRPC6. One theory is that sarcolemmal membrane instability in DMD renders these channels more susceptible to mechanical activation (46). Other factors include altered posttranslational modifications, including greater phosphorylation by Ca^2+^ calmodulin–dependent kinase (CaMKII) (47) or extracellular response kinase (ERK1/2) (48) both activating TRPC6, or depressed phosphorylation by protein kinase G, which normally inhibits TRPC6 (49). TRPC6 conductance and expression are also increased by oxidative stress (16, 50, 51) found in DMD with mechanostimulation (24, 52). While no oxidized residues on TRPC6 have been found, ROS could indirectly enhance conductance by oxidizing protein kinase G (53) or CaMKII (47).

TRPC6-mediated Ca^2+^_i_ is unlikely to directly affect muscle excitation-contraction coupling, as the quantity of Ca^2+^ conducted by TRPC6 is very small (nM) relative to cyclical Ca^2+^ transients (μM) (49, 54). That said, it is possible this impact is altered by DMD, and that remains to be studied. While chronic suppression may have an effect, prior work in myocytes from the *Trpc6-*KO mouse does not support such changes (4). In contrast, TRPC6 has the potential to regulate cell signaling, as TRPC6-mediated Ca^2+^ has been linked to profibrotic signaling (55, 56) involving TGF-β, p38, and CN/NFAT (56). ERK1/2 is also stimulated by TRPC6 activation (57, 58), though whether this is adverse or beneficial is less clear (59).

The transcription factor and disease Ingenuity pathway analysis found that TRPC6 small-molecule blockade particularly modifies metabolic pathways that would downregulate lipogenesis and control overall lipid and glucose utilization as well as suppress profibrotic signaling. Direct confirmation of these effects and the effect of metabolic changes remain to be elucidated. It should be noted that these data are from the heart; whether similar patterns are found in skeletal muscle remains to be determined. That there are both substantial differences and similarities between the TRPC6 gene deletion and drug inhibition conditions is not surprising, given that one involves embryonic deletion, the other postnatal suppression. As mentioned, the small-molecule inhibitor can also affect other proteins in a TRPC6 heterotetramer and so differentially alter transcriptomics.

Limitations of this study include the use of DKO mice lacking both dystrophin and utrophin; humans with DMD do not share the latter deletion. This still provides a model that better captures the severe DMD phenotype found in humans. TRPC6 inhibition does not fix the underlying cause of DMD, yet its efficacy despite this highlights its relevance to the pathobiology. To test clinical utility in treating DMD, TRPC6 antagonists will likely be studied in patients with DMD who also are treated with steroids and perhaps other therapies aimed at restoring functional dystrophin. Whether the drug therapy is impactful in this setting remains to be tested. Genetic strategies have generally targeted specific mutations, meaning that many patients are not candidates and, even if successful, there may still be value in suppressing TRPC6. Finally, we would comment on the varied sample size among assays. This was largely due to the fragility of the DKO model, precluding each measure being made in each mouse. Many DKO mice succumbed prior to a particular scheduled assay. Thus, measurements and the cohorts in which they were obtained were assembled over time to assure age-matched comparisons. These factors led to the different sample sizes, but all available data for a given assay are presented. Our study also does not identify the downstream mechanisms by which TRPC6 inhibition improves DKO and HET DMD mice. Many cell types could be involved, and this will require more detailed studies in the future.

The moderate sample size used in our study, typical of many similar animal studies, means we cannot rule out that small differences with borderline *P* values in either direction may be subject to false-positive or false-negative error. However, we viewed our findings as suggestive when observed differences for a given outcome/assay were in a similar direction and size to other independent assays addressing similar behavior. The design of the current study aims was to focus on clear contrasts and large effects, contrasting them to areas where no statistically significant differences were found to further shed light on the relevant biology. Small changes due to TRPC6 inhibition might be missed, but we believe in the setting of the controlled design used here; such smaller differences are likely less important to the overall biology than larger ones and mortality benefits, which were highly significant.

In conclusion, we identified TRPC6 as a major component of the pathophysiology of DMD — showing that its chronic suppression genetically and, most importantly, using a small-molecule inhibitor improves striated muscle function, improves bone remodeling, lessens muscle fibrosis, and improves survival in one of the most severe DMD mouse models yet generated. Ongoing development and clinical testing of BI 764198, a related TRPC6 inhibitor, may pave the way for studies in individuals with DMD.

## Methods

### Experimental design.

The breeder, HET, and *mdx/utrn^+/–^/Trpc6^–/–^* strains used to generate DKO, HET, and TKO mice were previously backcrossed into the C57BL/6J strain (39). Litters from the breeder strains were alternated between treatment groups to ensure that effects of treatments were not due to the effect of specific breeders. Mice were fed ad libitum and supplemented with hydrogel upon weaning. BI 749327 dosing was started on P3 at 30 mg/kg/d administered by subcutaneous injection (up to 200 μL in volume) until their natural demise or terminal study. Controls were injected with vehicle only (methylcellulose solution: 2.5% methylcellulose in deionized water by weight/volume with 0.015% Tween 80). The 30 mg/kg/d dose was determined from pharmacokinetic studies performed previously in various background strains ensure appropriate exposure for daily dosing (21). While BI 749327 is orally bioavailable, this molecule cannot be mixed in food or dissolved in drinking water without monitoring intake in individual mice, and oral gavage was not feasible in the fragile mice. All animals were monitored daily for health. Experimental measures were taken throughout the study until a predetermined endpoint or mortality, which limited availability of mice for all experiments. All measurements were age matched at the time of study ±1 week and performed by operators from treatment groups. Experiments and analysis were similarly conducted in a blinded fashion.

### MicroCT of femurs.

High-resolution images of the mouse femurs were acquired using a desktop microtomographic imaging system (Skyscan 1272, Bruker) (60) in accordance with the recommendations of the American Society for Bone and Mineral Research (61). Bones were scanned at 65 kV and 153 μA using a 0.5 mm aluminum filter with an isotropic voxel size of 10 μm. In the femur, trabecular bone parameters were assessed in the 500 μm proximal to the growth plate and extending for 1.5 mm (150 CT slices). Femoral cortical bone structure was assessed in a 500 μm region of interest centered on the middiaphysis.

### Open-field mobility test.

Mice were placed in a 40 cm × 40 cm box, and their movements were tracked by an overhead camera for 30 minutes and analyzed using AnyMaze software (Stoetling). Total distance traveled, average speed, and percentage of time in the center (as defined as middle 50% of the open field) were recorded.

### Forelimb grip strength.

Mice were placed onto a metal grid attached to a grip strength meter (Harvard Apparatus). With only their forelimbs gripping the metal grid, the mice were gently pulled by the base of their tail until they released their grips. The force (g) was recorded, and the grip strength test was measured in triplicates, the average of which was used per mouse per measurement.

### Rearing behavior.

Mice were moved from their home cage into a clean cage, and video recordings were taken for 1 minute after they were moved into their new environment. Recordings were manually scored blind for rearing behavior, as defined as when the mouse raises up vertically on its hind limbs.

### Skeletal muscle function.

Muscle function was measured in situ using a 305C-FP muscle lever system (Aurora Scientific Inc.), as previously described (62). Anesthetized mice (isoflurane, to effect) were placed on a thermostatically controlled platform. The knee of the mouse was secured, and the foot of the mouse was firmly fixed to the footplate of the torque sensor. Needle electrodes were inserted behind the knee in proximity to the sciatic nerve to obtain isometric contractions from the gastrocnemius muscle. Optimal isometric twitch torque was determined by increasing the current. A gap of 30 seconds minimum was used between each contraction to avoid fatigue. Peak isometric tetanic torque contractions (0.2 ms pulse, 250 ms train, 150 Hz) were measured by taking the peak of 3 successive contractions, with 3-minute gaps in between contractions. Mice were allowed to rest for 5 minutes. The susceptibility to eccentric contraction damage was examined with 20 sequential tetanic contractions (100 Hz, 0.2 ms pulse width, 250 ms duration), and at 200 ms, the muscle was rapidly stretched at a rate of 40 degrees per second through a 30-degree angle. The muscle was then allowed to returned to resting length. Maximal force of the isometric plateau (prior to the eccentric stretch) was measured and used to normalize the force decrease. The maximal force of the last tetanic contraction was divided by the first tetanic contraction to determine relative eccentric injury.

### Echocardiography.

Transthoracic echocardiography was performed in conscious mice (Vevo 2100, VisualSonics) as previously described (39). Briefly, conscious mice were assessed using M-mode modalities, using an average of 3–5 cardiac cycles. Heart rate, LV dimensions, and wall thicknesses were used to derive the measurements presented. The operator was blinded to animal treatment.

### Tissue collection and histology.

Heart weight, lung weight, body weight, and tibia length were recorded upon sacrifice of an animal at the terminal point of the experiment. A midtransverse cross-section of the heart encompassing both the LV and right ventricle was dissected using a heart slicer matrix. Transverse sections of skeletal muscles (gastrocnemius, soleus, tibialis anterior, and diaphragm) were all excised tendon to tendon where possible. All tissues were fixed overnight in SafeFix II (Fisher Scientific), followed by paraffin embedding, sectioning of 2 transverse sections per animal, H&E and Masson’s Trichrome staining, and imaging at ×20 (Aperio ScanScope CS). Cross-sectional area of muscle fibers was manually determined in a blinded manner. The entire transverse section was also analyzed in a blinded manner for percentage fibrosis, expressed as percentage of total area using 2 independent programs: Aperio ImageScope macro (Leica Biosystems Imaging, Inc.) and ImageJ (NIH). The remaining myocardium was flash frozen in liquid nitrogen and stored at –80°C.

### Plasma levels of BI 749327.

Plasma samples were analyzed by liquid chromatography–tandem mass spectrometry upon protein precipitation. The API 5000 triple-quadrupole mass spectrometer (AB Sciex) was operated in the positive ion mode. The lower limit of quantification was 1 nmol/L. Pharmacokinetic parameters were calculated by means of noncompartmental analysis from the plasma concentration-time curves as previously described (63).

### RNA-Seq analysis.

mRNA isolation and RNA-Seq was conducted by Genewiz. Total RNA was isolated from flash-frozen heart tissues of DKO mice, BI 749327–treated DKO mice, or TKO mice (*n =* 3 biological replicates, age and sex matched). mRNA was enriched by poly-A selection and configured for 150 bp paired-end reads. Sequencing depth approximated 350 M reads per sample; these were filtered for a sequencing quality of at least Q30. Differential gene expression analysis was performed with DESeq2, using the gene count output from RSEM. The top differentially expressed genes (FDR < 0.05) were used for principal component analysis (R) and pathway analysis (IPA, Qiagen). Processed data, counts, analysis, and code are publicly available on Github (https://github.com/skannan4/trpc6-dmd; branch, main; commit, 712c723; 624a36e; c2f5da0; and 2e8fd63).

### Statistics.

All statistical analyses were conducted using GraphPad Prism 9.3.0. Mantel-Cox log-rank tests were conducted for Kaplan-Meier survival curves. Two group comparisons employed 2-tailed Student’s or Welch’s *t* test (the latter if group variances significantly differed) or Mann-Whitney *U* test (if nonnormally distributed). Data were plotted as mean ± SD, with exact *P* values provided. A *P* value of equal to or less than 0.05 was considered statistically significant, and *P* values are labeled regardless of significance. We did not perform a prestudy power analysis, as the effect size was unknown, but we did perform a post hoc sample size calculation using α = 0.05 (false positive) and 1-β = 0.2 (false negative) based on the measured means and group variance. This was met in more than 70% of the comparisons. For comparisons with borderline significant or trending *P* values based on a *P <* 0.05 cutoff, the sample size was suitable at 60% to 70% power using α = 0.05.

### Study approval.

All of the mice in this study were maintained in accordance with Johns Hopkins University IACUC–approved procedures, and the protocol was approved by the Johns Hopkins University Animal and Care Use Committee.

## Author contributions

BLL, WPDJ, CL, OH, and S Kwon performed the primary experimental protocol, including histological and morphological studies. JS analyzed the mouse RNA-Seq data. NW performed and analyzed the echocardiography. S Kannan analyzed human RNA-Seq data. MCM and RCR conducted and analyzed microCT analyses. EV performed microCT imaging of the spine. CWW performed and analyzed force-frequency and eccentric injury studies. SSP and AF assisted in study design and pharmacokinetic analyses. BLL and DAK designed the study, analyzed the results, and wrote the manuscript. DAK was responsible for the project overall.

## Supplementary Material

Supplemental data

Supplemental table 3

Supplemental video 1

Supplemental video 2

## Version 1 09/13/2022

In-Press Preview

## Version 2 10/10/2022

Electronic publication
