# Supplementary material for: Pharmacological TRPC6 inhibition improves survival and muscle function in mice with Duchenne muscular dystrophy
Source: JCI Insight. 2022 Oct 10;7(19):e158906. doi: 10.1172/jci.insight.158906 (PMC9675567; doi:10.1172/jci.insight.158906)
Supplement: Supplemental data [file jciinsight-7-158906-s245.pdf]

Supplementary Materials

- Supplementary Figures and Legends
- Supplementary Movie
- Supplementary Tables 1-2
- Supplementary Table 3 is provided as an Excel spreadsheet on line.

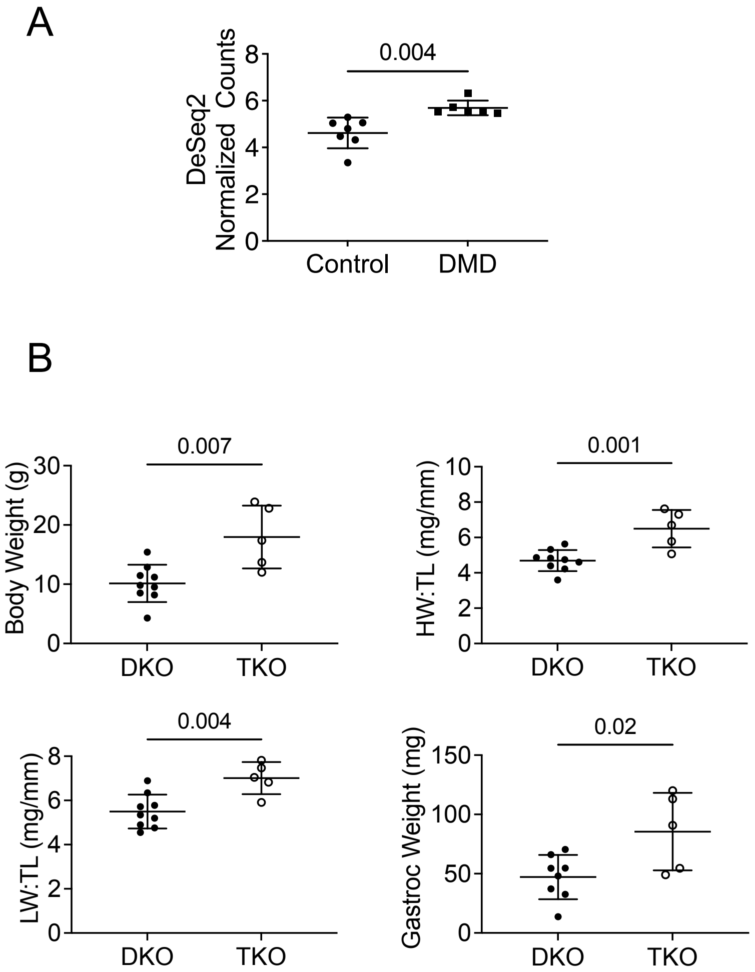

Supplemental Figure 1. TRPC6 is elevated in human DMD, and DKO (mdx/utrn$^{-/-}$) with additional deletion of *Trpc6* (TKO) have increased body, heart, lung, and skeletal muscle weight. (A) Analysis of published RNA-Seq data from reported FASTQ sequencing files (24) of skeletal muscle biopsies from human DMD patients shows increased TRPC6 expression. P-value form Pearson T-test. **(B)** Body weight, heart weight/tibia length (HW:TL) lung weight/tibia length (LW:TL), and gastrocnemius (Gastroc) weight measured in DKO versus TKO mice (n=9 DKO, n=5 TKO, p values from Pearson's T-test).

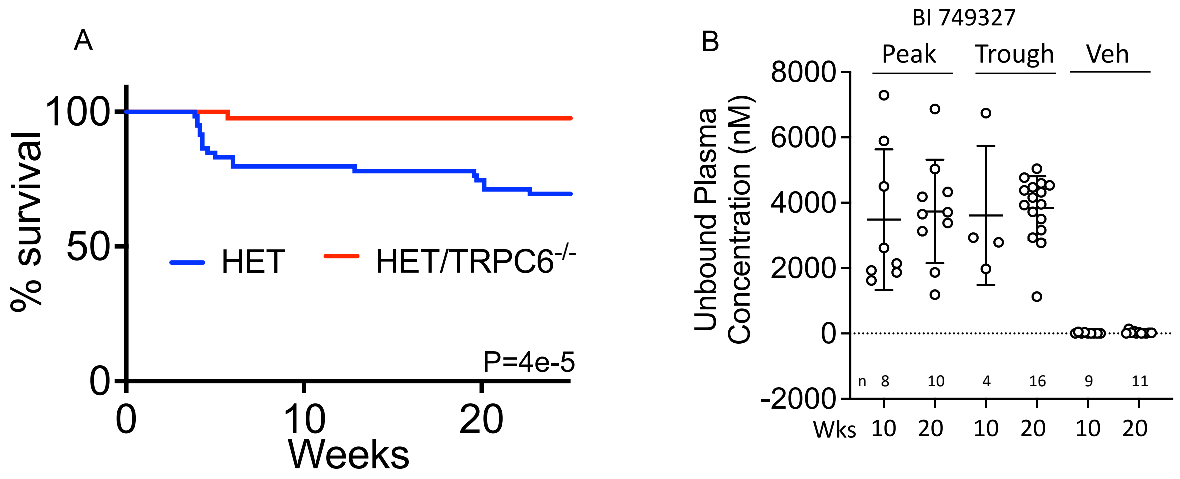

Supplemental Figure 2. TRPC6 deletion prolongs survival in unperturbed HET mice, and the exposure of BI 749327 in HET mice. (A) Survival curves in HET (*mdx/Utrn*$^{+/-}$) mice and those lacking *Trpc6* (HET/*Trpc6*$^{-/-}$) (Log-rank test, n=59 HET, and n=41 HET/Trpc6$^{-/-}$). **(B)** Free plasma BI 749327 concentration in HET mice with 30 mg/kg/day dosing over 10-20 weeks. Blood was obtained by cardiac puncture at terminal study 2-hours post-dosing (peak) 24 hours post-dosing trough). The median concentration was 2-4 mM. Sample size for each measurement provided in figure. Brown-Forsythe ANOVA shows no significant difference in dose levels at either time point (P=0.97). Drug is not detected in vehicle-only treated mice.

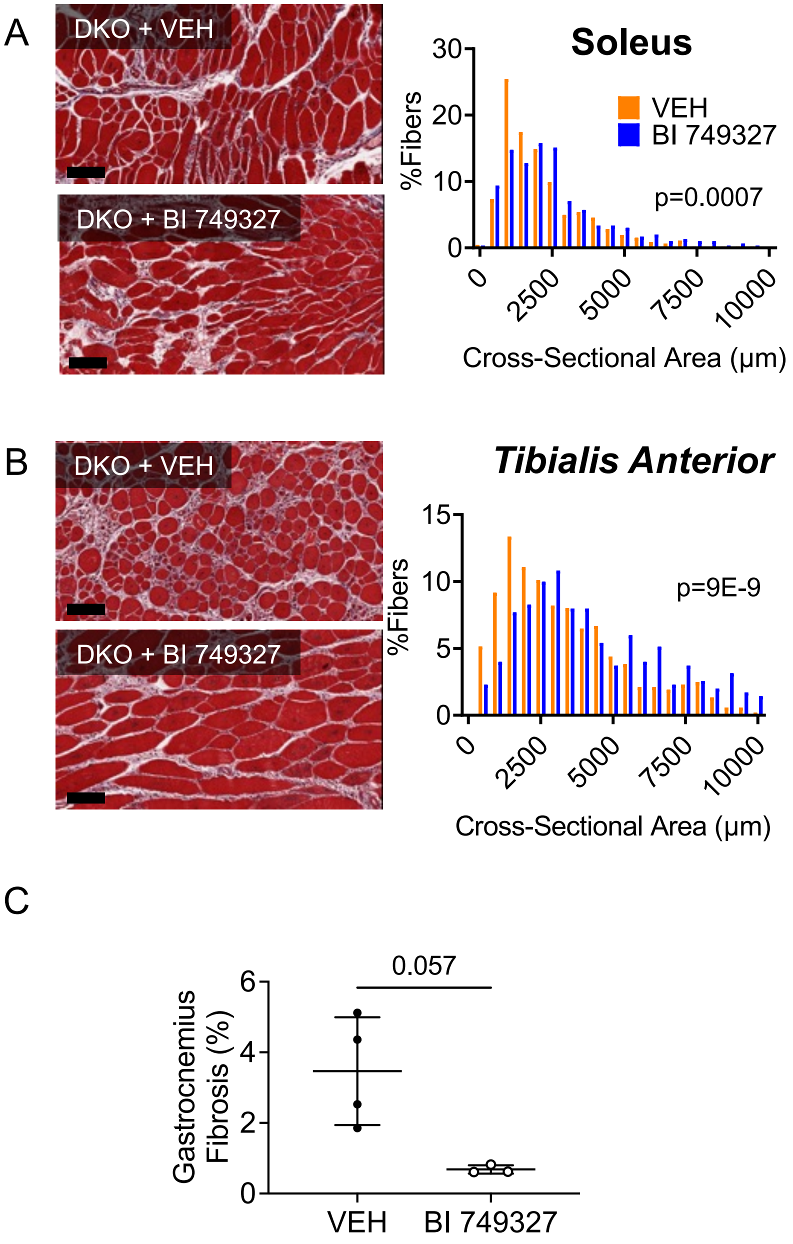

Supplemental Figure 3. BI 749327 treatment in DKO improved histopathology. Example histology (Masson's Trichrome staining) of **(A)** soleus and **(B)** tibialis anterior (TA) skeletal muscle, and summary data for fiber cross-sectional area in mice treated with vehicle (placebo) or BI 749327 (soleus: n=464 vehicle, n=298 drug fibers; tibialis anterior: n=524 vehicle, n= 351 drug fibers; from n=3 animals/group, Analysis by Mann-Whitney test). Scale bar = 100 μm. **(C)** Percent fibrosis from whole transverse sections of gastrocnemius muscle (n=4 vehicle, n=3 drug; Mann-Whitney test).

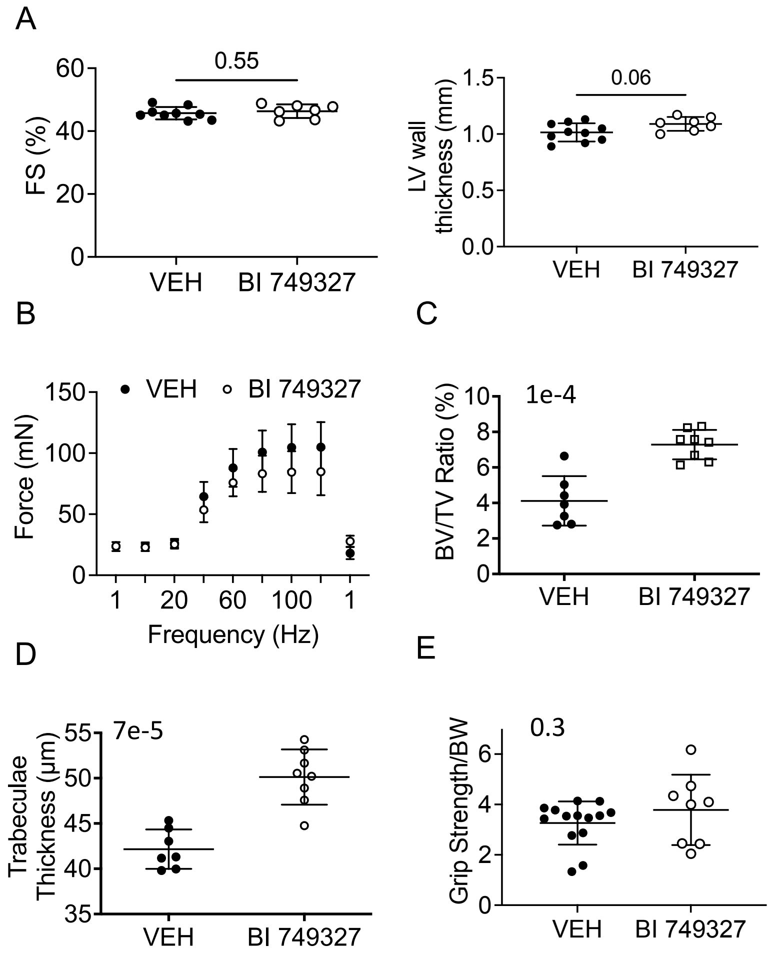

Supplemental Figure 4. BI 749327 treatment in HET mice does not increase striated muscle function in adulthood.

(A) Echocardiography measurements in HET mice showed no significant change in fractional shortening or LV wall thickness at 13 weeks of age. The full set of parameters are provided in Supplemental Table 2C. **(B)** In situ gastrocnemius contraction at varying frequency and post peak contraction - at rest. Muscles in mice at 20 weeks of age from each treatment group. (n=5 vehicle, n=4 BI 749327). **(C)** Bone volume to tissue volume ratio and **(D)** trabeculae thickness in from 13-week HET (n=7 vehicle, n=8 BI 749327). **(E)** Grip strength in HET +/- BI 749327 normalized to body weight shows no difference between groups. All P values displayed are from Student’s unpaired T test.

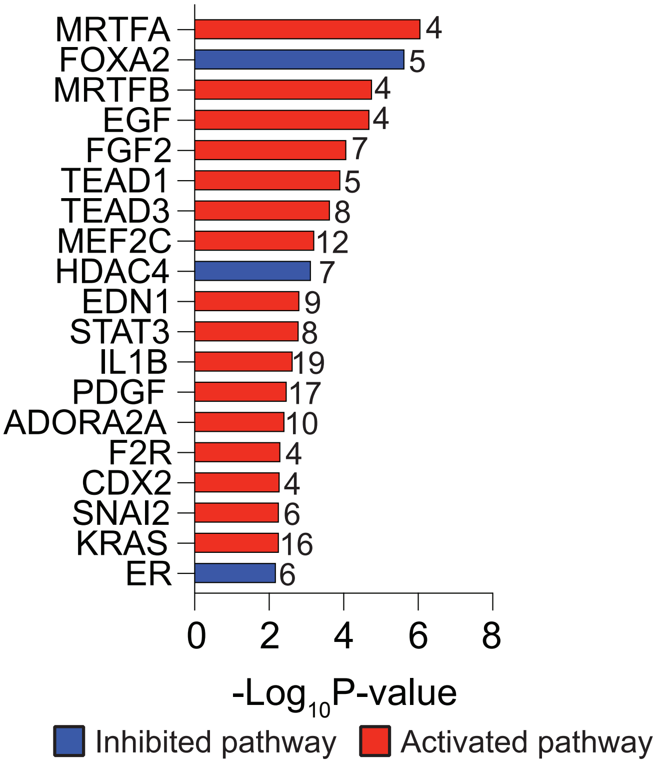

Supplemental Figure 5. Transcriptome comparison of DKO vs. TKO genes. Ingenuity pathway analysis of proximal gene signaling pathways found in the genetic comparison (DKO v TKO) upstream regulators for differentially enriched genes (all with absolute activation z-score > 2).

Supplementary Movie:

DKO Mice (*mdx/utrn*$^{-/-}$) are video-imaged routinely walking about in their cages. On the left are mice that were treated with placebo (vehicle treated), and on the right are mice treated with the TRPC6 antagonist - BI-749327 (drug treated). Those receiving active treatment display greater mobility and rearing (rising up on their hind legs).

Supplementary Table 1. Comparison of bone and motor function in DKO-vs-TKO study, and DKO +/- BI 749327 Study. Analysis by Kruskal-Wallis and Dunn's multiple comparisons vs. WT controls, unless otherwise specified (*one-way ANOVA).

Table 1A: Comparison of bone and motor function in DKO and TKO versus WT control mice.

	**WT**			**DKO**				**TKO**			
8-weeks of age	Mean	STD	n	Mean	STD	n	p-value vs WT	Mean	STD	n	p-value vs WT
BV/TV Ratio	17.3	7.6	5	5.19	0.82	5	0.06	8.39	2.12	4	0.06
Trabeculae Number (mm^{-1})	2.84	0.67	5	1.32	0.185	5	0.008	2.00	0.43	4	0.29
Trabeculae Spacing (µm)	189.8	17.3	5	295.7	19.03	5	0.008	225.7	24.96	4	0.06
Distance (m)	61.55	16.4	6	11.47	7.5	14	0.00007	34.72	5.20	5	0.5
Speed (m/s)	0.034	0.009	6	0.006	0.041	14	0.00008	0.019	0.003	5	0.5
Time in Center (%)	7.1	2.01	6	5.0	2.26	14	0.39	15.47	4.86	5	0.14
Grip Strength (g)*	130.3	22.37	6	41.85	16.56	11	1.31E-5	79.03	15.1	11	0.056

Table 1B: Comparison of motor function in DKO+vehicle (VEH) and DKO+BI 749327 versus WT control mice.

	WT			DKO+VEH				DKO+BI749327			
6-weeks of age	Mean	STD	n	Mean	STD	n	p-value vs WT	Mean	STD	n	p-value vs WT
Distance (m)	61.13	3.22	3	39.96	11.81	6	0.0455	55.52	16.47	7	0.74
Speed (m/s)	0.034	0.0017	3	0.021	0.007	6	0.042	0.031	0.009	7	0.76
Grip Strength (g)	125.2	8.48	3	40.98	12.59	5	0.0035	55.13	12.85	10	0.07

Supplementary Table 2. Cardiac morphology and function in three experimental groups (also with WT control comparisons). Data are mean and standard deviation. P values are T test with equal or different variances based on whether F-test confirms signficant variance disparities between comparison groups.

TABLE 2A: DKO versus TKO

		Age	LVID;d	LVID;s	Mean WT	EDV	ESV	SV	CO	EF	FS	HR	Geometry
		weeks	mm	mm	mm	µL	µL	µL	ml/min	%	%	BPM	
DKO	mean	7.64	2.87	1.84	0.67	31.41	10.54	20.87	8.93	66.75	35.75	432.7	0.23
(n=7)	sd	0.63	0.15	0.20	0.05	4.03	2.95	2.66	1.17	6.88	5.01	72.0	0.02
TKO	mean	7.55	3.03	1.95	0.89	36.35	12.68	23.67	13.11	66.64	36.13	551.0	0.30
(n=7)	sd	0.29	0.28	0.39	0.07	8.08	6.55	3.50	3.38	10.69	7.65	90.8	0.03
WT Control	mean	7.90	2.53	1.10	0.81	22.90	2.65	20.25	14.18	88.41	56.51	700.4	0.32
(n=5)	sd	1.09E-15	0.023	0.02	0.14	0.51	0.13	0.53	1.00	0.63	0.88	43.5	0.05
DKO vs TKO		0.74	0.22	0.57	**0.0001**	0.21	0.48	0.15	**0.028**	0.98	0.92	0.03	**0.003**
DKO vs Con		0.36	**0.002**	**0.0002**	0.1977	**0.003**	**0.001**	0.71	**0.0003**	**0.0005**	**9.7E-05**	**0.001**	**0.007**
TKO vs CON		**0.03**	**0.006**	**0.003**	0.426	**0.009**	**0.01**	0.06	0.50	**0.0041**	**0.001**	**0.03**	0.40

TABLE 2B: DKO +/- BI-749327 Treatment

		Age	LVID;d	LVID;s	Mean WT	EDV	ESV	SV	CO	EF	FS	HR	Geometry
		weeks	mm	mm	mm	µL	µL	µL	ml/min	%	%	BPM	
DKO-vehicle	mean	5.71	2.65	1.80	0.70	26.26	10.17	16.09	8.67	62.33	32.29	534.86	0.27
(n=7)	sd	0.71	0.29	0.29	0.07	6.15	3.40	3.28	3.01	7.02	4.80	131.92	0.04
DKO-BI 749327	mean	6.03	2.65	1.42	0.82	26.13	5.41	20.72	12.00	79.57	46.38	586.29	0.31
(n=7)	sd	1.17	0.29	0.18	0.08	7.27	1.81	5.55	2.82	2.38	2.25	96.85	0.04
WT Control	mean	6.36	2.60	1.42	0.89	24.73	5.32	19.40	13.21	78.58	45.34	680.67	0.34
(n=5)	sd	0.05	0.14	0.11	0.06	3.34	1.17	2.40	1.74	2.31	2.16	24.27	0.01
ttest DKO +/- BI-749327		0.56	0.97	**0.012**	**0.015**	0.97	**0.007**	0.08	**0.05**	**0.0004**	**0.00001**	0.42	0.07
ttest DKO-veh v con		**0.053**	0.73	**0.02**	**0.0008**	0.63	**0.008**	0.08	**0.01**	**0.0005**	**0.0002**	**0.03**	**0.004**
ttest DKO drug v con		0.48	0.76	0.99	0.16	0.70	0.93	0.63	0.42	0.49	0.44	**0.04**	0.13

TABLE 2C: HET +/- BI-749327 Treatment

		Age	LVID;d	LVID;s	Mean WT	EDV	ESV	SV	CO	EF	FS	HR	Geometry
		weeks	mm	mm	mm	µL	µL	µL	ml/min	%	%	BPM	
HET-Vehicle	mean	13.39	2.98	1.69	1.01	34.71	8.61	26.10	15.96	75.53	43.49	611.80	0.34
(n=10)	sd	0.85	0.19	0.27	0.08	5.21	4.24	4.79	2.93	9.72	7.27	33.56	0.04
HET-BI-749327	mean	13.66	2.88	1.55	1.09	31.95	6.70	25.24	13.75	79.27	46.34	557.71	0.38
(n=7)	sd	0.05	0.24	0.17	0.06	6.84	2.06	4.93	1.60	2.35	2.18	90.95	0.04
ttest-drug +/-		0.34	0.33	0.24	**0.054**	0.36	0.29	0.73	0.09	0.27	0.27	0.10	**0.054**
